# Supplementary figures and images for: Evaluating the Impact of Intraoperative MRI in Neuro-Oncology by Scientometric Analysis
Source: Life (Basel). 2022 Jan 25;12(2):175. doi: 10.3390/life12020175 (PMC8877236; doi:10.3390/life12020175)

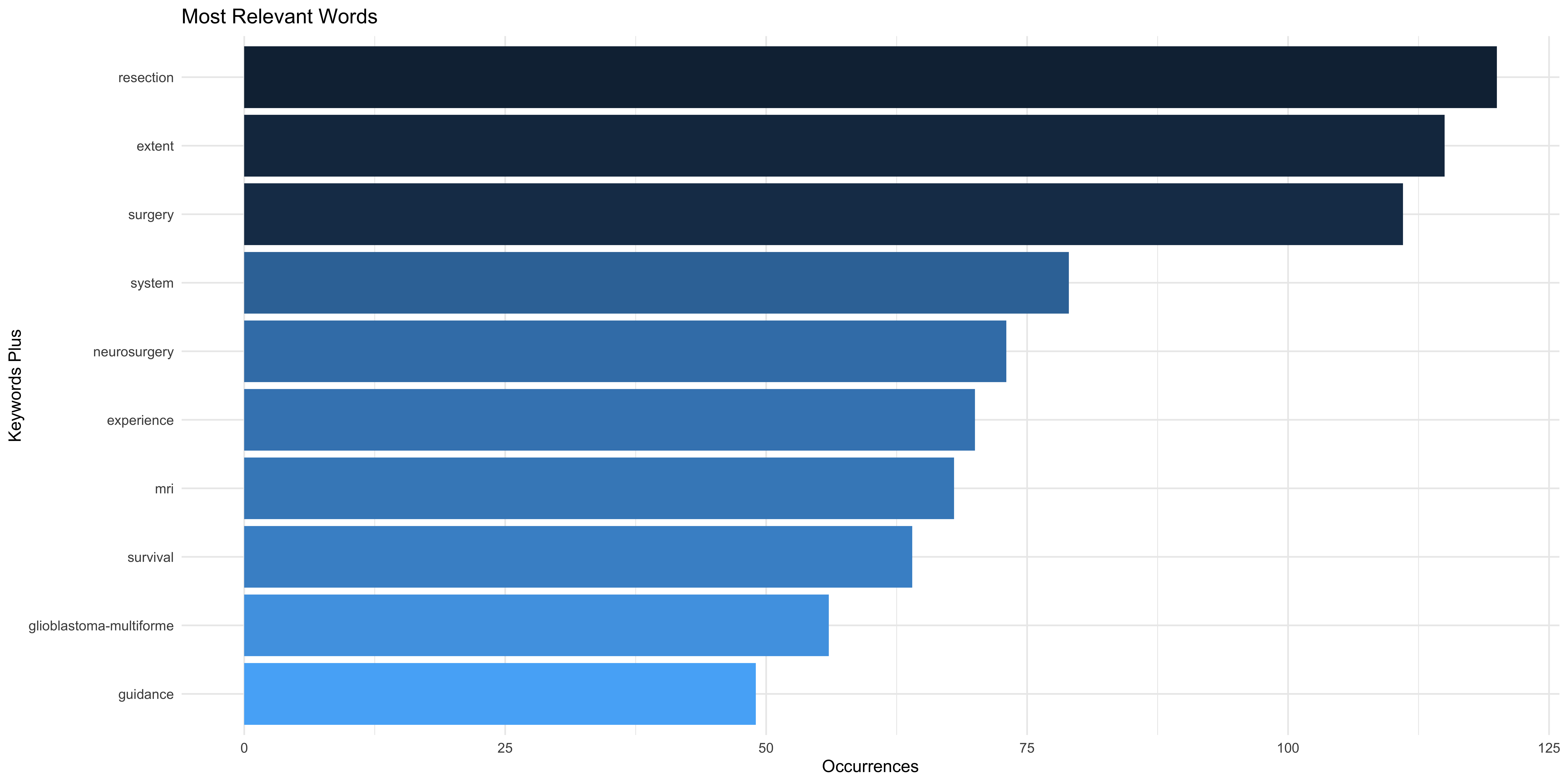

Supplement: Supplementary file 1 [file life-12-00175-s001.zip › life-1525074-supplementary/Supplementary figure S1.png]

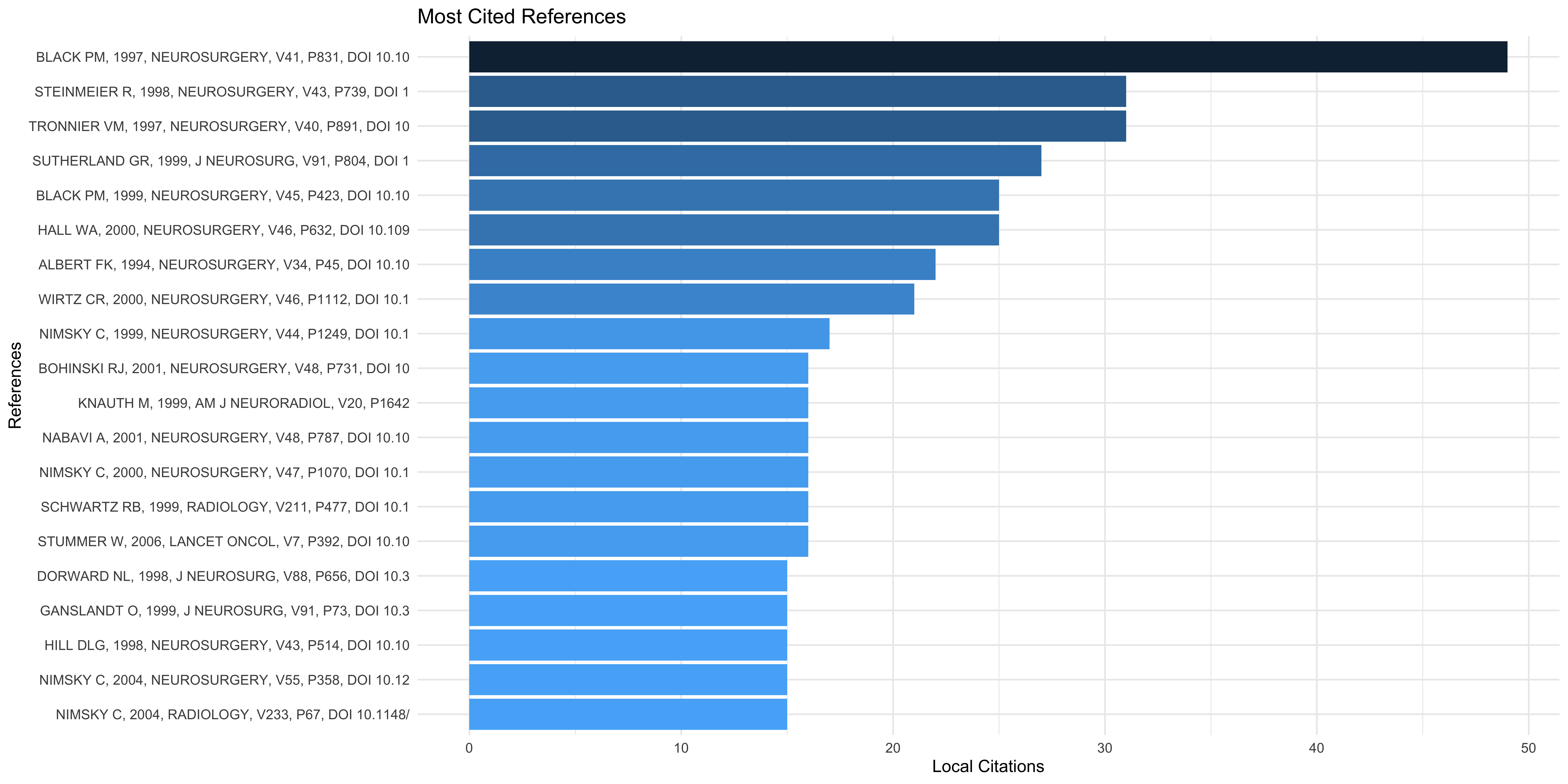

Supplement: Supplementary file 1 [file life-12-00175-s001.zip › life-1525074-supplementary/Supplementary figure S2.png]

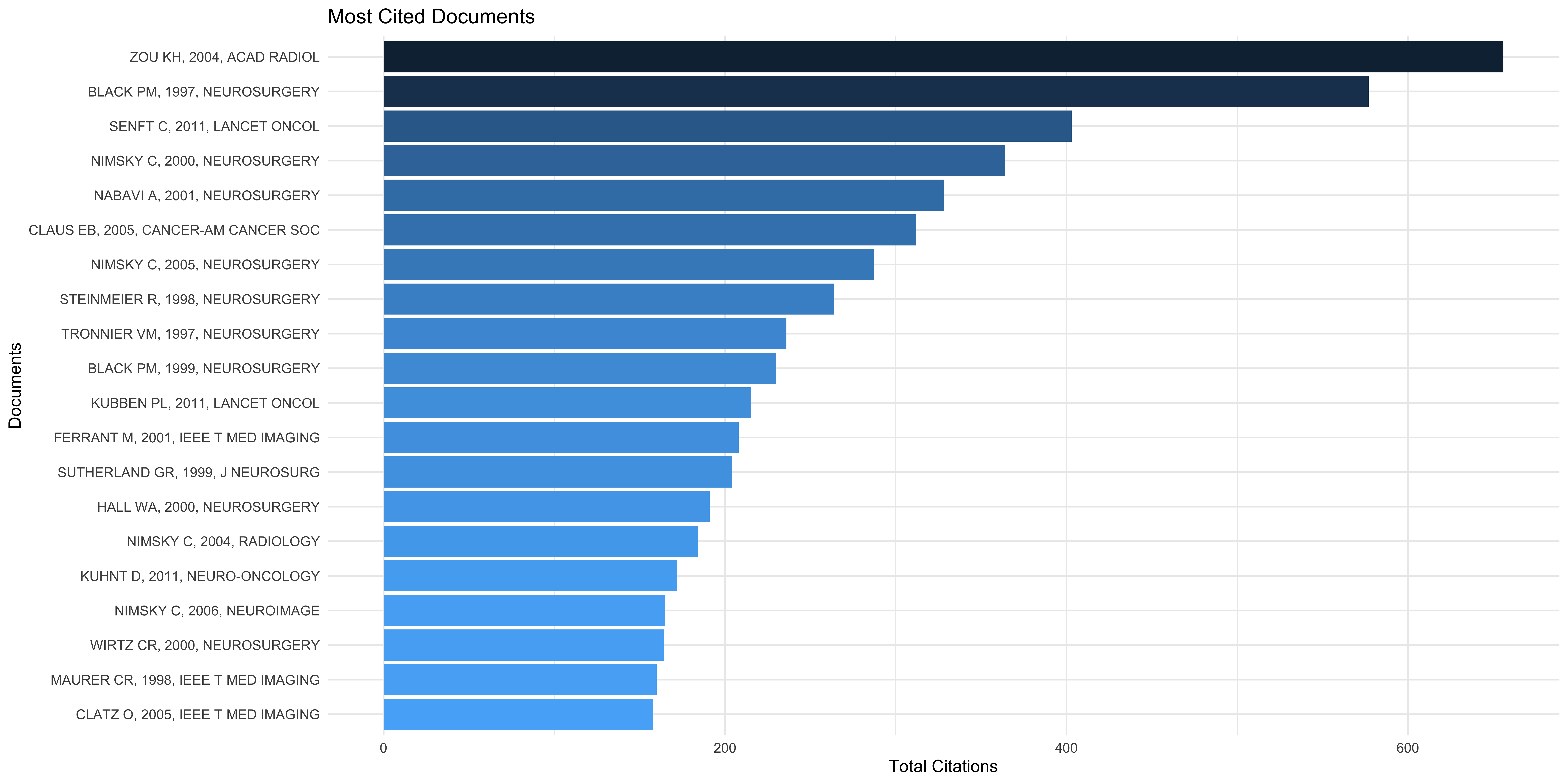

Supplement: Supplementary file 1 [file life-12-00175-s001.zip › life-1525074-supplementary/Supplementary figure S3.png]
